# Freedom, diseases, and public health restrictions

**DOI:** 10.1111/bioe.13217

**Published:** 2023-08-28

**Authors:** Alberto Giubilini

**Affiliations:** ^1^ Wellcome Centre for Ethics and Humanities, Oxford Uehiro Centre for Practical Ethics University of Oxford Oxford UK

**Keywords:** COVID‐19, freedom, health, lockdown, pandemic, public health

## Abstract

The debate around lockdowns as a response to the recent pandemic is typically framed in terms of a tension between freedom and health. However, on some views, protection of health or reduction of virus‐related risks can also contribute to freedom. Therefore, there might be no tension between freedom and health in public health restrictions. I argue that such views fail to appreciate the different understandings of freedom that are involved in the trade‐off between freedom and health. Grasping these distinctions would allow to appreciate why different people give more weight to different aspects of limitations of freedom, including whether certain options are made simply risky or impossible, whether limitations of freedom are posed intentionally or happen accidentally, whether risks are beyond a threshold of acceptability, and who gets to decide that. I provide a conceptual analysis of the relationship between different types of freedom, public health policies, viruses and diseases. As I argue, identifying what freedom‐based reasons count for and against different types of public health restrictions requires distinguishing between viruses and diseases, between lockdowns and other types of restrictive policies, and between risks posed by viruses and threats of penalties involved by restrictive policies.

## 1 INTRODUCTION

Lockdowns and other restrictive pandemic policies were often seen as ways of trading off individual freedoms—including civil liberties—for public health.^1^ However, health and reduction of risks posed by viruses also constitute dimensions of freedom. For instance, more options are open to you when you are not sick. Given the central value of freedom and civil liberties in liberal societies, a crucial question is about which type of freedom should be prioritized when implementing public health policies. Answering this question requires some conceptual clarity about how different understandings of freedom are involved in the trade‐off between freedom and health. In this article, I intend to provide a conceptual map of the tension between freedom and health and between different types of freedom that are affected by restrictive public health policies, viruses, and diseases. I aim to clarify which reasons are available and which ones are not available to those who want to make a case for lockdown and for other types of public health restrictions on grounds of protecting freedom in the long term. Identifying such reasons requires properly distinguishing between viruses and diseases, between lockdowns and other types of restrictive policies, and between risks posed by viruses and threats of penalties involved by restrictive policies.

Before getting to the core of this discussion, it is useful to look at three possible ways of understanding that trade‐off between individual freedom and health in restrictive public health policy.

First, freedom and health might be taken to be incommensurable. When I say that two things are incommensurable, here, I follow Ruth Chang's notion of incommensurability and mean that they ‘cannot be precisely measured by some common scale of units of ‘value’.^2^ This is more or less what we mean when we say that someone is comparing apples and oranges. Incommensurability *could* be taken to imply that freedom and health cannot be compared for the purpose of ranking them by ethical importance.

Second, freedom and health, even if incommensurable, might still be compared and ranked for the purpose of determining which one should take priority in public policy. On some views,^3^ incommensurability does not imply incomparability. Sometimes, we can compare and rank incommensurable things according to their relative importance, even if we cannot quantify by how much one is better than the other. That is, even if freedom and health cannot themselves be measured according to a common scale, their respective values might. You might still think one is ethically more important than the other on the basis of some intuition you have, for instance.

Third, health and freedom might be taken to be commensurable, because they do have a common scale of units of value.

The third interpretation is the most desirable one, as it would make the weighing of freedom against health just a matter of computational comparison of gains and losses of the same unit of value. The third one grounds the views of those who challenge the idea that there is a trade‐off between freedom and health in restrictive public health policies. On this view, both lockdowns and viruses restrict freedom by preventing people from doing the things that they would like to do. This view has often been implied in public health communication during the last pandemic^4^ and has recently been defended in the academic literature.^5^

I will argue that the relationship of viruses and of lockdowns with freedom is more complex than this commensurability view suggests. That level of complexity has ethical and political implications that we might end up overlooking if we think that viruses and lockdowns restrict the same freedom. While *some* aspects of freedom limitations caused by viruses and by lockdowns are commensurable, others are not. In particular, if we take lockdowns as the benchmark for limitation of *negative* freedom, then viruses, to the extent that they limit freedom, do not limit negative freedom in the same sense and in the same way as lockdowns do.

If, instead, we take other types of public health restrictions (say, a limit on the number of people who can gather, as has often been the case during the first year of the recent pandemic) as the benchmark for limitation of negative freedom, I will argue that the freedom that is restricted might well be the same as the one that viruses restrict, so the third interpretation above might be available: viruses and public health restrictions constrain the same type of freedom. However, the way freedom is restricted in the two cases is different and the differences have ethical and political implications for the justification of restrictive policies.

## 2 VIRUSES AND LOCKDOWNS: THE SAME FREEDOM AT STAKE?

Let us start with an analysis of the claim that lockdowns and viruses restrict the same type of freedom. Kieran Oberman has recently argued for this claim. In his article, Oberman refers to former U.S. President Donald Trump's statements that invoked the language of freedom to oppose lockdowns in certain U.S. states, as well as statements by anti‐lockdown protesters that also referred to lockdowns as an unjust restriction on freedom. Oberman thinks that this approach is mistaken. He believes that ‘[t]he problem with Trump and the anti‐lockdown protesters is […] that they do not fully understand the implications of the value they profess to love’. This is because, in Oberman's view, both viruses and lockdowns can restrict the negative freedom that we all enjoy when there are no significant external constraints posed by others. Such constraints include both viruses carried by vectors (i.e, persons) and state‐imposed restrictions. On this view, lockdowns can promote negative freedom by keeping viruses under control in the longer term. Thus, on his view, negative freedom does not necessarily count as a reason against lockdowns. In Oberman's view, if lockdowns are necessary to keep viruses under control, and if uncontrolled viruses would sufficiently limit our freedom by causing significant disease or death, then lockdowns might actually increase our negative freedom overall by protecting us from viruses. Besides, lockdowns might also reduce unequal distribution of freedoms by restricting those who already enjoy sufficient freedom from disease in order to promote the freedom of those who are more vulnerable.^6^

I am going to suggest that the main problem with Oberman's arguments is the failure to give due consideration to two distinctions. One is the distinction between viruses and diseases, which prevents him from seeing that one of the freedoms that ‘Trump and the antilockdown protesters’ care about is the freedom to take risks, rather than having the State decide what risks they should be allowed to take. One could criticize their view, of course, but it is important to understand first what their view is, and it seems Oberman misses the target here.

The other distinction is between freedom from physical constraints and freedom from psychological constraints, such as fear, risk aversion, or other psychological barriers. I will address these two conceptual problems in Sections 3 and 4, respectively.^7^

Before analysing these problems, a few notes on definitions. I accept Oberman's characterizations of lockdowns as ‘any nontargeted measure enforcing social distancing’ and that of viruses as ‘any contagious pathogen, whether technically a virus or otherwise, that is so dangerous that a government might consider a lockdown in response’. The definitions are far from perfect from a technical point of view, but they capture well the conceptual and ethical issues that lockdowns and viruses raise when it comes to the way they restrict freedom.

I also accept Oberman's characterization of ‘negative freedom’ as the freedom that ‘can only be restricted by (1) external constraints (2) imposed by other people’.^8^ This aligns with Isaiah Berlin's original definition of negative freedom as ‘the degree to which no man or body of men interferes with my activity’.^9^ For the sake of argument, I also assume, with Oberman, that viruses can be external limitations of freedom posed by other people, in the sense that they ‘external in source, but not in location’,^10^ and that the source is humans acting as vectors.

Finally, here, I will refer mostly to SARS‐CoV‐2 virus, COVID‐19 disease, and lockdowns, because they provide the most recent and obvious examples of the tension between freedom and health. However, Oberman’ claims are broader in scope and do not apply only or necessarily to COVID‐19, but extend to other infectious diseases. In the same way, my points are generalizable to other comparable public health threats, different from infectious diseases.

## 3 VIRUSES VERSUS DISEASES, POSSIBILITIES VERSUS COMPOSSIBILITIES

Viruses can sometimes cause diseases. For example, the Severe Acute Respiratory Syndrome Coronavirus 2 SARS‐CoV‐2 *sometimes* causes the disease COVID‐19. *Sometimes*, however, the same virus does not cause any disease. Even before there were high levels of ‘natural immunity’, asymptomatic cases of SARS‐CoV‐2 infections were estimated to be around 40% of all infections,^11^ and many symptomatic cases are relatively mild. Young people are significantly less likely to develop significant symptoms.^12^ Asymptomatic cases are not cases of disease, though they are cases of infection with the virus.

The distinction is crucial to appreciate the different ways in which viruses and diseases affect different types of freedom. The following passage from Oberman seems to overlook that distinction:

Just as lockdowns place people under threat of being fined for leaving home, so viruses place people under threat of infection. That threat prevents the conjunctive exercise of the freedom to perform actions that lead to infection and the freedom to perform actions that infection prevents. While, subjectively, infection is no certainty, we can again make sense of risk by seeing matters objectively. There are locations where virus particles are present. These locations represent points in spacetime where people cannot go and perform certain actions without being infected^13^

Here, Oberman is adopting Ian Carter's and Mathew Kramer's view whereby you are unfree to do things when it is impossible, and not merely costly for you to do them.^14^ Oberman says that viruses' threats remove freedom because they "prevent" you from doing certain things, that is, they make those things impossible for you. If you go to the pub and catch a virus and get very sick, you are then unfree to leave home, in the same way as you would if you were under a lockdown. But if this is the understanding of freedom that we assume, it seems that Oberman is downplaying the difference between viruses and diseases, and therefore the role of the probabilistic nature of the threat posed by viruses (and virus‐carriers) for the purpose of identifying limitations of freedom.^15^ It is true that if I develop a serious disease, it means that I am not free to perform both the action that leads to the disease (say going to the pub where I caught the virus) *and* the actions that infection has prevented (say, leaving my home once I got sick). But the conjunction of the exercise of the two freedoms is not prevented by the mere *threat* of infection, that is, by the presence of a virus. It is only prevented by an infection resulting in a serious enough disease. Yet, the virus might not result in any infection and an infection might not result in any disease.

More specifically, it is simply not true that there are locations where ‘people cannot go and perform certain actions without being infected’, even if virus particles are present. There are locations where it is more or less likely that someone gets infected, but not locations where someone will necessarily get infected.

There are two senses in which we can understand the term ‘location’ here. In neither sense is that claim true.

In a broader sense, consistent with common language, ‘location’ refers to any place identified by the criteria with which we commonly divide the space into places, such as shops, rooms, restaurants, train coaches, and so forth, *within* which the virus is present. Different locations will have higher or lower probabilities of infection—some of them will have very high probabilities—but no such location is a location where a person *will* necessarily get infected.

In a narrower sense, very detached from common language but that might offer more support to Oberman's claim, the relevant ‘location’ is the minuscule space occupied solely by each single viral particle. If people's noses or mouths happen to be in this exact location, they will breathe the virus in. However, even in this sense of the term, it is not true that people will necessarily be infected in that specific location. They might have enough natural immunity from previous infection with SARS‐CoV‐2. They might have T‐cells that developed from previous infections with other viruses. Or they might have enough immunity from a recent vaccination. In all such cases, one's immune system would react and prevent infection even if someone has breathed the virus in.

So, on either understanding of ‘location’, we are simply talking about a risk of infection with higher or lower probability. But if probabilities of infection, or of any other kind of risk, were sufficient to undermine the freedom to perform actions that come with these risks *and/or* the actions that infection prevents, then it is hard to see how the notion of freedom that Oberman uses here can be meaningful. Basically anything carries with it *some* risk to ourselves or to others, or more often to both. Driving a car carries with it the risk of accidents that could kill us and/or kill others, or leave us and/or others disabled. So, it seems that Oberman would be forced to say that the risk of car accidents reduces or even eliminates our freedom because whenever we drive, it is possible that we have an accident whose consequences will reduce or eliminate our future freedom. That is not consistent with the way the notion of freedom is commonly used—if anything, we would say that driving expands our freedom. Indeed, it would make the concept rather meaningless.

As for the second element of the ‘conjunctive exercise’ of freedom, namely, ‘the freedom to perform actions that infection prevents’, how would a viral infection itself restrict such freedom? Here, Oberman is following Matthew Kramer's account of limitation of negative freedom, whereby negative consequences of my actions can limit my future freedom by foreclosing future possibilities^16^ (including, e.g., the option of performing joint actions with the persons who will die from catching a virus from me). Once again, viruses are not diseases. Asymptomatic individuals would not have their *negative* freedom restricted. The same is true for those with mild enough symptoms. For all I know, I could be infected right now even if I am feeling very well, or I might catch a virus from my colleague who is about to knock on my office door. However, since I do not have any symptom and in case I do not develop any symptom after my colleague infects me, I am free to come to the office today and all the following days and to go to the pub after work. I am free in the sense that there is no constraint imposed by another person (such as a human vector of the virus) that prevents me from doing that. It is only if my viral infection turns into a disease that my negative freedom would be constrained by the disease.

True, if I know that I am infected or that people around me are infected, I might have *moral* constraints, for example, if I thought I have the responsibility to try not to get infected and not to risk infecting others. Or I might have *psychological* constraints, for example, if I am very risk‐averse and I want to minimize chances of getting infected. But these constraints do not limit the same freedom that a disease limits. Surely, a plausible notion of negative freedom needs to be consistent with the freedom to be reckless or morally irresponsible—assuming for the sake of argument that I would be reckless or irresponsible by coming to the office.

Now, Oberman does not refer to these two types of actions—the actions that lead to infection and the actions that infection prevents—individually, but as a conjunction. He claims that both viruses and lockdowns deprive us of the possibility to do *both* things. To quote him again, ‘[t]hat threat [posed by viruses] *prevents* the conjunctive exercise of the freedom to perform actions that lead to infection and the freedom to perform actions that infection prevents’ [emphasis added]. We have just seen that the threat does not prevent them individually, because viruses are not diseases. Therefore, a fortiori, it does not prevent their conjunction either. It merely makes their conjunction less probable.

Oberman refers to the conjunction of the two because he is drawing largely on accounts of negative freedom defined in terms of compossibilities, that is, sets of options, rather than possibilities, that is, individual options. Two things are compossible if they are ‘possible in combination’.^17^ An example of compossibility is the possibility of going out *and* not dying from a virus that I caught while going out.

Compossibilities provide a more meaningful measure of freedom than mere possibilities, because they are a measure of how many options I can keep open or I foreclose by doing something or refusing to do something, such as going out or staying home during a pandemic. The more compossible actions I have, the more overall freedom I have. Overall freedom is ‘the amount of freedom one has […] and represents some kind of an aggregation over one's specific freedoms’.^18^ The notion of overall freedom as used here is mathematical in nature. In principle, you can calculate how much overall freedom individuals have by adding up the compossibilities available to them.

What we have said so far implies that viruses make compossibilities, but not possibilities, less likely. The fact that viruses entail merely risks of future constraints means that they preserve the freedom to choose certain possible actions, like going to the pub, and therefore the freedom to take risks. However, being risks, they reduce the chances that compossibilities are available—if I become very sick from catching a virus at the pub, or if I die from it, I will not have the freedom to *also* go on holiday the next week. Thus, by reducing the likelihood of compossibilities, viruses reduce the likelihood that you will have as much overall freedom as you have in their absence. This also applies to the asymptomatic: in their case, viruses reduce the chances that they will be able to associate with those whom they might infect. And it applies to the immune, too, in case they want to associate with someone who is not immune and might get the disease. In this sense, it is true that viruses reduce (overall) negative freedom, but even assuming that the one just used is a plausible notion of negative freedom, it is not the type of negative freedom that lockdowns restrict. Lockdowns differ from viruses in two relevant ways.

First, they restrict possibilities, and not just compossibilities, so they target specific freedoms. Second, they restrict them by making options impossible, not just less likely ‐ so the restriction is a total one: negative freedom is taken away, not simply reduced in proportion to risks. I will expand on these two points in the next section. I will argue that *if* lockdowns are the standard with which we measure limitation of negative freedom, then viruses do not restrict the same understanding of negative freedom as lockdowns do. We might still use the term ‘negative freedom’ to refer to both, provided that we bear in mind that they are two substantially different sub‐types of negative freedom. One (viruses) has to do with reduced probability of future actions and the other (lockdowns) with immediate impossibility of actions. The difference has relevant ethical and political implications for our assessment of public health restrictions, because to some people, the freedom to take risks matters a lot.

## 4 VIRUSES VERSUS LOCKDOWNS: RISKS VERSUS IMPOSSIBILITIES

Oberman refers to lockdown and viruses as ‘threat of being fined’ and ‘threat of infection’, respectively, as if they both shared the same probabilistic nature and therefore were equivalent in terms of restrictiveness. But that is a mistake. Lockdowns are not merely threats. They make certain actions impossible, not just costly and less likely. Oberman writes that ‘even when threats are enforced, they do not make noncompliance impossible. During a lockdown, people can leave their homes. They might be fined, but they can still leave’.^19^ This is a very limited view of lockdown and the freedoms involved. There is more to freedom than being free to be outside your home. Indeed, most of the times, you leave home to do more meaningful things. These are the things that lockdown makes impossible, not just costly or risky. Lockdowns are structural society‐wide restrictions. If pubs are closed, you do not have the freedom to go to the pub even if you are willing to risk the fine. If travels to a country are banned, you do not have the freedom to travel even if you are willing to ignore the travel ban. If businesses have to temporarily or permanently close, you do not have the freedom to go to work or, in some cases, to do the things that having an income allows. If schools are closed, children do not have the freedom to go to school and parents the freedom to keep on working normally. Quite simply, there are no pubs open, no planes operating, no workplace for the ones that cannot work online, schools are shut, and so on. And, of course, these restrictions take away many other freedoms that presuppose the freedoms just mentioned, such as the freedom to socialize in certain places, the freedom to attend funerals for communal mourning of loved ones, and so on.

Someone might object that lockdowns are not universally enforced. For instance, if the government orders that all pubs be closed, some pubs might violate the order. Or if the government closes the border, some people will enter the Country illegally. So going to the pub or travelling during a lockdown are also a matter of taking risks, as in the case of being exposed to viruses. One can expose oneself to the risk of legal penalties and therefore of being subject to limitations of freedom in the form of jailtime of heavy fines. In the same way, exposing oneself to a virus is a matter of taking the risk of being subject to limitation of freedom in the form of a bad disease or of death. This is an objection worth considering, but I am not convinced by it. It seems that it ignores the ways in which lockdowns are designed and enforced. The point of a lockdown is to target a whole societal system and with that the networks that make options possible. A lockdown implies, by its very own nature, a structural shutdown whereby the impossibility of many options is embedded in the design of the policy. After all, many options are normally available to us only to the extent that there is a sufficient level of societal functioning, which lockdowns remove. A single pub might well violate a lockdown order, but that would just create a state of non‐lockdown locally and only for as long as the wider societal shutdown does not prevent the pub from getting supplies. ‘Going to the pub’ would still be impossible to most of the population under the lockdown. It would only be possible, subject to the acceptance of risk of penalty, to the few people who live in the illegal non‐lockdown area. That is because when we normally say that we are free to go to the pub, or to travel, and so on, we are referring to actions with certain reasonable spatio‐temporal and practical constraints, which we normally do not need to make explicit. For instance, when I say that I want to go to the pub, or to travel abroad to visit my family, I am not saying that I want to go to any pub even if it is a hundred miles away, or that I am equally happy to travel hidden in a freight train. A lockdown might still preserve the freedom to do these things, but that would not be the freedom to go to the pub or to travel as we normally understand them. At best, these are surrogates of those freedoms. If a minimum level of societal functioning is not in place, the real options are made impossible. It is only if enough components of society were to break lockdown rules and preserve a sufficient level of coordination and functioning that my freedom to go to the pub or to travel would be preserved—precisely because these options presuppose the existence of a sufficient level of societal coordination and functioning. There would be flights operating illegally, with the required adequate staff working illegally; there would be pubs open illegally thanks to a supply chain operating illegally. At some point policing such violattion would become unfeasible. But this, quite simply, would not be a state of lockdown.

Having said so, I am happy to concede that some aspects of lockdown are indeed susceptible to the objection. A stay‐at‐home order, for instance, seems to be a case where you can just take the risk of legal penalties for doing exactly what the order prohibits you from doing, that is, stepping outside of your house. I am happy to concede this point. This means that some aspects of lockdowns are comparable to more common public health restrictions, which simply come with the risks of penalty. I am going to discuss these in Sections 7 and 8, where I point out that even in those cases, there are relevant differences in the way viruses and public health measures restrict my freedom, even when the type of freedom restricted is the same (negative).

Relatedly, it is worth noting that even if both viruses and some aspects of lockdowns restrict negative freedom, there are other types of freedom at stake that some aspects of lockdowns and viruses restrict differently. Most obviously, for instance, you are not *politically* free to do something if the only way for you to do it is doing it illegally. Thus, some aspects of lockdowns undermine such *political* freedom in the way in which viruses do not, even if they both also restrict negative freedom. Again, I will return to this point in Sections 7 and 8.

If my arguments above are correct, then lockdowns do not restrict freedom in the same way as viruses do. They do not target compossibilities by foreclosing options attached to certain actions in the way viruses might do; instead, they target those actions directly. And they target them by making them impossible, rather than just less likely. If we consider both these aspects, then lockdowns could restrict freedom in the same way as very serious diseases do, but even then, there are important differences to consider. Lockdowns restrict freedom for a significant amount of time for the vast majority of people. On the contrary, a disease like COVID‐19, if it restricts freedom, only restricts it for very few days for the vast majority of people. Death and serious illness from viruses would restrict negative freedom much more than lockdowns do, of course. But again, they are a matter of probability.

In any case, lockdowns could have long‐term debilitating effects on individuals’ health as well. These include, to mention just a few, an increase in the number of anxiety disorders, including eating disorders,^20^ as well as the socioeconomic impact on people living on a low income or hand‐to‐mouth food.^21^ All these consequences could have a larger impact on freedom than the virus would. An eating disorder, for instance, is likely to have a lifelong impact on a person's behaviour.

## 5 LOCKDOWNS AND POSITIVE FREEDOM

Now, what I have said so far does not imply that viruses do not restrict freedom. It only implies that they restrict freedom differently from the way lockdowns do. This is compatible with two claims. One is that viruses restrict a different type of freedom from lockdowns. The other is that both viruses and lockdowns restrict the same freedom, the negative one, but a different variety of it. That is, viruses restrict the same variety of negative freedom that more common restrictive public health policies restrict. I am going to discuss the first view in this section, followed by some considerations of its ethical and political implications in Section 6. In Section 7, I will discuss the second view, again followed by some ethical and political considerations about it.

The extent to which threats posed by viruses or vectors prevent individuals from doing things that might lead to infection depends on how willing individuals are to take risks. If someone is afraid of catching a virus to the point of not wanting to associate with others to avoid risks, then the barrier is psychological, not external.

Importantly, to say that it is psychological is not to say that it is not a barrier to freedom, or that it is less of a barrier than an actual disease or other injuries or harm. Fear might well be a rational and fitting response to a threat. At this stage, I am only concerned with the nature of the barrier and of the freedom restricted, not with a prudential, ethical, or political evaluation of it.

A large number of people—and not only the most risk‐averse—would not have their freedom constrained by their attitudes to risks if risks were relatively low. That is, in conditions of relative security. The lower the risks, the more even the most risk‐averse will feel free to do the things they want to do. Necessary and sufficient conditions for security might be difficult to pin down. Arguably, security as an objective state of affairs (e.g., as defined by certain probabilities of bad outcomes) would need to be associated with an adequate perception of it, so that people can feel safe enough to pursue their goals.^22^ In any case, it seems that on any plausible account of it, ‘security implies a stable, relatively predictable environment in which an individual or group may pursue its ends without disruption or harm and without fear of such disturbance or injury’.^23^ This seems to be a precondition—necessary but not sufficient—for the freedom to pursue one's valuable goals in life.^24^ Thus, freedom from viruses is the freedom that we have when we are enabled to pursue our goals, values, and, ultimately, our self‐realization by having reasons to feel secure enough. These are some of the elements that can be found in many accounts of positive freedom,^25^ defined by the presence of certain enabling conditions that make me, so to speak, my own master when it comes to making decisions for myself. In the words of Isaiah Berlin, the positive sense

is involved in the answer to the question ‘What, or who, is the source of control or interference that can determine someone to do, or be, this rather than that? […] [t]he positive sense of the word ‘liberty’ derives from the wish on the part of the individual to be his own master. I wish my life and decisions to depend on myself, not on external forces of whatever kind.^26^

To the extent that it gives us reasons not to fear risks beyond a certain level, security allows us not to be dominated in our decisions by our fears and the conditions that generate them—such as threats posed by viruses. If the threat posed by the virus is very large, people might not *feel*, and therefore *be* free in the positive sense, even if they are free in the negative sense.

The relationship between negative and positive freedom is traditionally problematic in many respects, such as whether they are actually different freedoms^27^; which one represents the more genuine form of freedom^28^; and which one should be promoted by liberal Governments.^29^ For the purpose of the present discussion, I merely want to point out that viruses can limit positive freedom by introducing risks that undermine people's (sense of) security.

## 6 DIFFERENT FREEDOMS: ETHICAL AND POLITICAL DISAGREEMENTS

The different types of freedom that lockdown and viruses restrict are at the core of significant ethical and political disagreement around a couple of questions.

One question is axiological, which is about the relative value of negative and positive freedom: which one is more valuable? For some, positive freedom is the only meaningful understanding of freedom. As Charles Taylor put it, for instance, ‘[y]ou are not free if you are motivated, through fear, inauthentically internalized standards, or false consciousness, to thwart your self‐realization’.^30^ For others, such as Amartya Sen, it is the most valuable one because it consists of the possession of the relevant capabilities that allow one to live a fulfilling life.^31^ Negative freedom does not differentiate among valuable and nonvaluable options: you are equally free in the negative sense regardless of whether the absence of human interferences allows you to do something meaningful or something trivial. Positive freedom, instead, retains the distinction: you are not free in the positive sense if you do not have the freedom to do what really matters to you. Ethically and politically, to many, it makes a difference which freedom is targeted through government actions or inactions.

This leads to a deontic question, which is a question about duties regarding protection of freedom: which type of freedom does a state, at least in liberal democracies, have an obligation to prioritize, and in what way? If the distinction between negative and positive freedom gets lost in the claim that the same type of freedom is at stake when we talk of viruses and of lockdowns, we would not be able to understand why people with different ethical and political views disagree on lockdowns.

In this respect, the distinction between negative and positive freedom seems to track another distinction with ethical and political relevance. That is the distinction between active state intervention, which typically promotes positive freedom through, for example, subsidization, welfare programs, redistributive taxation policies, or restrictions on individual actions to reduce risks to others; and a minimal state that does not intervene, except to protect individuals against undue force, fraud and theft.^32^ State intervention often causes some level of harm or infringement of negative freedom for some for the sake of benefiting or redistributing resources to others, so as to promote their positive freedom. A minimal state would allow more harms and inequalities to occur in society without interfering, thus prioritizing negative freedom. The former approach is the one that would justify lockdowns, which come with the certainty of reduction of the range of options available to individuals but also of reduction of risks from viruses (at least in the short term); the latter is the one that would leave people free to take their risks with regard to viruses.

As we saw, Oberman claims that ‘Trump and the antilockdown protesters […] do not fully understand the implications of the value they profess to love’, because they do not realize that a virus like SARS‐CoV‐2 can take away the same type of freedom that they think lockdown infringes. But if my analysis is correct, maybe—whether or not they are right in their ethical and political take on lockdown—they do understand at least something that seems to have escaped Oberman's analysis. This is the fact that lockdown makes certain actions impossible rather than just risky, and therefore takes away the freedom to take risks, which many people prefer (rightly or wrongly) over state impositions; and that with lockdown, we are infringing upon the negative freedom of the many for the sake of the positive freedom of the (relatively) few, which goes to the core of profound ethical and political divisions in society.

## 7 PUBLIC HEALTH RESTRICTIONS AND THREATS OF PENALTIES

Now, there are at least two challenges to my analysis so far.

The first is that lockdowns might not be too interesting a case for the purpose of a conceptual and ethical analysis of public health restrictions. More common public health restrictions (including pandemic ones) are unlike lockdowns in that they are based on threats of penalties. In this sense, one can choose to take the risks associated with breaking the law. When England had ‘the rule of six’ to limit the number of people who could gather during the COVID‐19 pandemic, for instance, you could have had a party of more than six and hope that you would not get caught. But it would be strange to claim that such coercive state interventions enforced through threats of penalties do not affect negative freedom, just because they come with the mere risk of suffering a penalty. And if it is true of restrictions based on risks of penalties that they limit negative freedom, it must be true of viruses and viral vectors as posing risks of disease or of death, too. Viruses would restrict negative freedom even if, unlike diseases, they merely involve some risk, for the same reason why many coercive public policies restrict negative freedom even if, unlike lockdowns, they merely involve some risk.^33^ Thus, there is a sense in which risks restrict my negative freedom, even if it is not the sense in which we say that lockdowns do when they make options impossible for me. But this is problematic, because as a matter of fact, so many things come with risks of future limitations of freedoms, for instance, in the form of injury, that we would be back to the problem of having to say that whatever we do limits our freedom.

The second challenge to my analysis is about the relationship between freedom and negative consequences of our free choices. The fact that certain behaviours come with negative consequences—whether legal penalties or death from viruses—does not prevent people from engaging in those behaviours. As Kramer rightly points out, ‘[u]sually, an addressee of a threat is able to perform the action which the threatener seeks to discourage’.^34^ This is also true in the textbook threat scenario, where someone is pointing a gun at you uttering the (credible) threat ‘your money or your life’. Even in that case, you are free not to hand in your money to the person with the gun, if you are prepared to die. Hillel Steiner has argued that threats of penalties for performing certain actions do not reduce our freedom, but merely change our preferences with regard to performing those actions.^35^ Yet, the actions are still available, and so we are as free to choose them as we were before the threat. Analogously, we saw earlier that viruses preserve the possibility to go out and risk being infected, even if they make certain compossibilities less likely. It is one thing to say that the consequences of my actions are such that I will lose my future freedom and it is quite another thing to say that I am not free to act in a way that will bring those consequences about. So also in this respect it seems that public health restrictions (other than lockdowns) and viruses are on equal footing with regard to freedom restrictions. Even if I knew for sure that by going to the pub I would get infected with a virus that will kill me, I would still be free to go to the pub. And even if I knew that by having a big party in my garden I will get caught breaking the ‘rule of six’ and go to jail or suffer financial penalties, I would still be free to have the party.

In the next section, I am going to address the two challenges that I have presented above, starting from the second one.

## 8 RISKS AND NEGATIVE FREEDOM

Ian Carter addressed a counterintuitive implication of Steiner's claim above—to recall, the claim that threats of penalties for performing certain actions do not reduce our freedom, but merely change our preferences with regard to performing those actions. The counterintuitive implication is that threats of penalties, which intuitively seem coercive and therefore freedom‐restrictive, would actually not make us less free. On this view, if you threaten me with ‘your money or your life’, or with ‘don't gather with more than 5 people or you will go to jail’, my freedom would be the same as the one that I had before the threat, and the only difference would be that my preferences (to keep my money or to see more than five friends at the same time) have changed. This seems implausible. Carter says that the way to avoid this implausible implication is, once again, by appealing to the distinction between *specific* and *overall* freedom.

From this point on, the story about how public health restrictions constrain my freedom is analogous to the story that I have told above about the way viruses restrict negative freedom. My freedom stays the same after the threat only in the *specific* sense of ‘freedom’. Threats of penalties preserve our specific freedom to act in the way that is legally punishable—which is the freedom that Steiner was referring to—but reduce our overall freedom because they make compossible actions less likely to be available to us. There are possible worlds where I can go out and avoid being fined or imprisoned. Luckily, most of us live in a world like this at the moment. And there are possible worlds where I cannot go out and keep my money or stay out of jail (depending on how rule breaking is punished). That is the world many of us lived in through the COVID‐19 pandemic. Threats of penalties simply mean that I am less likely to be able to engage in the behaviour that is legally sanctioned *and* not pay the cost in terms of the penalty that follows. Yet, I do have the *specific* freedom to engage in that behaviour—I would just need to accept the reduced likelihood of certain compossibilities.

We can therefore address the second challenge above by saying that, in fact, unlike lockdowns, neither viruses nor public health restrictions constrain our *specific* freedoms to go about our normal lives. We might just have to pay a cost for that—at the extreme, the cost is death or imprisonment—but we are free to choose that, in the same sense as, for instance, I am free to park my car in an unpermitted space and take the risk of receiving a parking ticket. However, to the extent that overall freedom is a function of the probability to have compossible actions available,^36^ both viruses and public health restrictions (other than lockdown) restrict our overall freedom.

Now, let us tackle the first challenge presented above. If risks impact negative freedom because they make compossibilities less probable, that will affect overall negative freedom, but so do many things that we normally do, including for instance driving a car. Yet, we do not say that we are made unfree, or overall less free, by driving or that driving reduces our freedom or our options—indeed, the opposite claim seems more plausible. Whenever I take the tube, there is a risk that some terrorist has placed a bomb in a station. Fortunately, such a risk is very small and to most of us acceptable. But it is not nonexistent. The same is true for viruses. There is always a risk that, by going about our normal daily lives, someone infects us with some bad virus, sometimes lethal ones. The seasonal flu is estimated to kill about 650,000 people worldwide every year,^37^ for instance. Again, many of us are lucky enough to live in a time and places where the risk is quite small for most people, though not nonexistent. It seems that in the case of an outbreak of a virus like SARS‐CoV‐2, such risks are increased. Similarly, when public health restrictions are introduced, risks of legal consequences are increased from zero (the risk before restrictions) to whatever probability there is that someone is caught engaging in prohibited behaviours. During the pandemic, hugging a friend was a risky activity for many because it could cost one a substantial fine. So how can we tell when our freedom has actually been restricted?

Even if our overall negative freedom is always restricted by the presence of some, however small, risk, it must be restricted over a certain threshold to allow us to *meaningfully* say that we are *not* free to do something or that we are not as free as we were before. The phrase ‘as free’ in the previous sentence is not to be read in a merely mathematical sense. It is about the meaning of freedom as we understand it in everyday language but also in ethical and political discussion. What makes a difference to whether our freedom is restricted is not the presence or absence of risks of deaths, disabilities, jailtime, or fines. These are always there. It is whether the risk is above or below a certain threshold that allows us to meaningfully say that our freedom is restricted. That threshold defines the risk both in terms of probability of the negative consequences and of the magnitude of such negativity. A very high risk of a very small penalty—say, a 5£ fine for breaking the rules of six or a very mild, short‐lived headache from a virus—might not infringe our freedoms, or at least not as much as a less likely, but far more serious consequence (say, jailtime or death).

Consider a law that is very poorly enforced. It does not meaningfully restrict overall negative freedom. In England, it is illegal to linger after a funeral or to pay with your phone at drive‐throughs while the engine is on. These laws are, needless to say, almost never enforced. As a consequence, not only do people have the specific freedom to do these things. They have the freedom of compossibilities too because they will always be free from the penalties attached to them. Such penalties restrict overall negative freedom only negligibly, given that the risks of penalties attached are very small. Hence, when we commit what would technically be a crime by lingering after a funeral, we are *as free*, overall, as when we leave straight after the service.

Or take again the car example. As said above, we would not say that driving reduces our overall freedom, just because it comes with risks of future freedom limitations—including, in the extreme, death. Not only do we have the freedom to drive despite the risks but also, when we drive, we are at least as free, overall, as when we do not drive (but have the option of doing so if we so wished), because the risk is considered normal or acceptable. ‘As free’ does not refer to the overall *quantity* of freedom. Rather, it means that we are free *in the same sense as* when we do not drive, other things being equal. That is because the risk of serious accidents that will limit my freedom remains below a certain acceptable threshold, the same threshold that allows us to say that we are meaningfully free.

A smallpox outbreak would reduce overall freedom very significantly by virtue of the very high risk of death involved. If there is an outbreak, it would probably make sense to say that our overall freedom is significantly reduced or has disappeared.

The key challenge here is that of identifying or agreeing on the threshold of acceptability, or of normal risks, after which we can meaningfully say that our overall freedom has been constrained by risks. I do not have an answer to this. It is an open question whether an outbreak of a virus passes that threshold and therefore reduces our negative freedom in a meaningful sense. It very much depends on the type of disease that the virus causes, of course. For the purpose of this discussion, suffice it to say that the simple claim that a virus comes with the risk of death is not sufficient to say in a meaningful way that the virus reduces our overall freedom to the same extent as public health restrictions do. At the same time, the presence of a *properly enforced* public health restrictions such as mask mandates, vaccine passes, limits to the number of people who can gather, and so on, certainly reduces our freedom significantly even if by introducing risks of penalty, rather than by making options impossible in the way lockdowns do. And the larger the penalty for the infringement, the greater the limitation of freedom. Public health restrictions like those that we experienced during the recent pandemic were not like the restrictions on lingering after funeral. For some of them, the level of enforcement was much higher.

## 9 RISKS VERSUS THREATS: AT THE CORE OF ETHICAL AND POLITICAL DISAGREEMENT ON FREEDOM

What we have said so far can be summarized as follows. Viruses restrict positive freedom and lockdowns restrict negative freedom. More specifically, lockdowns remove a certain variety of negative freedom, that is, the freedom that you have when certain possibilities are available to you. But as well as positive freedom, viruses can also restrict a different variety of negative freedom that also many public health restrictions (other than lockdowns) restrict. Both viruses and public health restrictions (other than lockdowns) can restrict a variety of negative freedom that is a function of the probability of having compossible actions available. Whether they restrict it to the same extent depends on the risk profile of the virus in question for different individuals, the level of enforcement of public health restrictions, and the severity of the penalties. Now, does this mean that public health restrictions and viruses restrict freedom *in the same way*? The answer is no.

Although the two terms are often used interchangeably (including in this article so far), for the purpose of the following discussion, it is useful to distinguish threats and risks. Threats are types of risks. More specifically, threats are risks of negative consequences, including restrictions of freedoms, that are intentionally posed by some human agent, including the State, when they threaten me with penalties for my behaviour. Threats are types of risk because the punishment that is threatened might not materialize. Risks more generally, that is, risks that are not threats, are posed unintentionally. That means that they might be posed by non‐human factors, like risks posed by floods or earthquakes, or they might be posed by human agents unintentionally, although knowingly.

Viruses pose risks, but not threats. Even assuming that the risk can meaningfully be posed by infected people, rather than by the virus itself, in most cases, it would be posed unintentionally, though not necessarily unknowingly. I might just want to go to the shops or to meet someone, but by doing this, I know that I might infect someone as an unintended consequence.^38^

Public health restrictions are not merely a risk. Someone has decided that the risk should be attached to my behaviour. That is, they are threats. The State has decided to threaten me with legal consequences for my behaviour.

We cannot simply assume that the intentional introduction of such risk is justified on the grounds of freedom as long it entails a net gain of overall negative freedom, that is, as long as the freedom that policies restrict is smaller than the freedom that an uncontrolled virus would restrict. The quality of that freedom is different even if the type of freedom is the same, that is, the negative type as defined by probability of compossibilities. Morally and politically, it matters how the reduction of freedom is brought about.

Morally, it matters because of the moral difference often attached to the distinction between doing and allowing. The idea that it is morally worse to cause a bad outcome (such as a limitation of freedom) than to simply fail to prevent the same outcome from occurring matches many of our common intuitions. It has been widely debated, with solid rejections^39^ and defences of it.^40^ For those who uphold this moral distinction, it makes a difference whether the limitation of freedom is simply something that an authority lets happen by not preventing a virus from spreading or something that the authority intentionally imposes through restrictive policies. Indeed, for some, it might be morally unacceptable to impose public health restrictions even if the freedom from viruses that would thus be preserved is larger than the freedom taken away by restrictions. That is because, on the assumption that viruses and public health restrictions do restrict the same type of freedom, one relevant question is not only how much of such freedom we can preserve but also *how* we can preserve it. On certain views, it is morally worse to have our freedom restricted by public health measures than by viruses. Or, to put it differently, we should tolerate the risks imposed by viruses—at least within certain limits—more than we tolerate the threats posed by the authority such as a State.

Politically, it matters how the reduction of freedom is brought about because that is the issue around which the disagreement over the legitimate scope of State intervention largely revolves. This takes us back to the very same political issue that we saw before when we discussed positive and negative freedom. Even if viruses make us in some sense less free, it might well be that the State has no business in addressing that kind of limitation of freedom—at least when addressing that requires introducing new freedom restrictions. It is one thing to suffer restrictions of freedom merely in terms of what is possible or likely available to us and it is quite a different thing to suffer restrictions of freedom in terms of what an authority allows us to do and the rights that it infringes—that is, restrictions of liberties.^41^

Or it might be that the State has a duty to address limitations of freedom imposed by viruses only when the risk to freedom posed by viruses is beyond a certain *ethically or politically relevant* threshold. Who decides what the threshold is and who decides that a certain virus’ risk is above that threshold are themselves ethical and political questions. What we said above about the political issues raised by the distinction between negative and positive freedom applies here as well. Those who lean towards the idea of a minimal State would set the bar for State intervention much higher than those who lean towards a more interventionist State.

## 10 CONCLUSIONS

Ethically and politically, what matters is not so much whether the same type of freedom is at stake, but the extent to which a State is justified in intervening to sacrifice a certain type of freedom for the sake of other types of freedom. Even assuming that viruses and public health restrictions constrain the same kind of freedom, that is, negative freedom, or indeed even assuming that viruses restrict that kind of freedom to a larger degree than policies do, it might be politically unacceptable to intentionally restrict freedom through threats of penalties, but acceptable to let a virus restrict freedom by posing risks. This is up for debate.

The issue is further complicated by the fact that different population groups might present different risk profiles for the same virus, and those who benefit from restrictions in terms of protection from the virus are not necessarily those who bear the highest costs of restrictions. Thus, there is a question about what counts as a fair distribution of (different types of) freedom and how to strike a balance between these two types of consideration.

While I have not addressed these substantive questions here, my discussion implies that a proper ethical and political assessment of lockdowns or indeed any other public health restriction is not simply a matter of a computational assessment of gains and losses of negative freedom. When it comes to viruses and to public health restrictions, it is important to identify and preserve the relevant distinctions between the different types of freedom involved, the different ways in which the same freedom can be restricted by viruses and public health policies, and the different magnitudes of such restrictions. This requires identifying the relevant differences between viruses and diseases, lockdowns and other types of restrictive policies, risks of diseases and threats of legal penalties.

Losing sight of such distinctions means failing to appreciate the different ethical and political issues that restrictive public health policies raise. It means failing to appreciate why many, including ‘Trump supporters and anti‐lockdown protesters’, react in a certain way to restrictive policies. Rather than being unable to understand the issues at stake, they are simply giving more weight to different aspects of freedom limitations. These include whether options are made simply risky or impossible, whether restrictions are intentionally posed or accidentally caused, whether risks are beyond a certain threshold of acceptability, and who gets to decide that. Disagreeing about the proper meaning and value of freedom in public health policy does not necessarily mean that one of the two parties does not understand freedom.

## Footnotes

1 See, for example, Gostin, L. O., Friedman, E. A., & Wetter, S. A. (2020). Responding to Covid‐19: How to navigate a public health emergency legally and ethically. *Hastings Center Report*, *50*(2), 8–12; Bennett, B., Freckelton, I., & Wolf, G. (2023). *COVID‐19, law, and regulation: Rights, freedoms, and obligations in a pandemic*. Oxford University Press; Ferdinand, K. C. (2021). COVID‐19 mitigation: Individual freedom should not impede public health. *American Journal of Public Health*, *111*(4), 592–593; Flood, C. M., MacDonnell, V., Thomas, B., & Wilson, K. (2020). Reconciling civil liberties and public health in the response to COVID‐19. *Facets*, *5*(1), 887–898; Bartolini, S., Sarracino, F., & Slater, G. (2020). *Do epidemics impose a trade‐off between freedom and health? Evidence from Europe during Covid‐19*. Munich Personal RePEc Archive. https://mpra.ub.uni-muenchen.de/105035/

2 Chang, R. (1997). Introduction. In R. Chang (Ed.), *Incommensurability, incomparability, and practical reason* (pp. 1–33). Harvard University Press. p. 2.

3 Ibid.

4 For instance, Italian Deputy Minister of Health defended lockdowns by saying that ‘having rigid rules today represents real liberty, normality comes with following the rules and not following them is contrary to future freedom’ (*The New European*, 25 September 2020). https://www.theneweuropean.co.uk/brexit-news-europe-news-johnson-freedom-remarks-criticised-by-italian-president-guardian-writes-94192/, accessed 15 July 2023.

5 Oberman, K. (2022). Freedom and viruses. *Ethics*, *132*(4), 817–850.

6 Ibid.

7 In his article, Oberman also discusses republican freedom as a kind of freedom that lockdowns and viruses might be taken to impact differently. However, his discussion there turns out to be more a criticism of the notion of ‘republican freedom’ than an argument for the equivalence of viruses and lockdowns. For this reason, and for reasons of space, I will leave that point out of the present discussion.

8 Oberman, op. cit. note 5, p. 825.

9 Berlin, I. (1969). Two concepts of liberty. In I. Berlin (Ed.), *Four essays on liberty* (pp. 118–172). Oxford University Press (Original work published 1969).

10 Oberman, op. cit. note 5, p. 826.

11 Ma, Q., Liu, J., Liu, Q., Kang, L., Liu, R., Jing, W., Wu, Y., & Liu, M. (2021). Global percentage of asymptomatic SARS‐CoV‐2 infections among the tested population and individuals with confirmed COVID‐19 diagnosis: A systematic review and meta‐analysis. *JAMA NetwOpen*, *4*(12), e2137257.

12 Li, B., Zhang, S., Zhang, R., Chen, X., Wang, Y., & Zhu, C. (2020). Epidemiological and clinical characteristics of COVID‐19 in children: A systematic review and meta‐analysis. *Frontiers in Pediatrics*, *8*, 591132; Ludvigsson, J. F., Engerström, L., Nordenhäll, C., & Larsson, E. (2021). Open schools, Covid‐19, and child and teacher morbidity in Sweden. *New England Journal of Medicine*, *384*(7), 669–671; Wang, B., Andraweera, P., Elliott, S., Mohammed, H., Lassi, Z., Twigger, A., Borgas, C., Gunasekera, S., Ladhani, S., Marshall, H. S. (2022). Asymptomatic SARS‐CoV‐2 infection by age: A systematic review and meta‐analysis. *medRxiv* 2022.05.05.22274697; Poletti, P., Tirani, M., Cereda, D., Trentini, F., Guzzetta, G., Sabatino, G., Marziano, V., Castrofino, A., Grosso, F., Del Castillo, G., Piccarreta, R., Andreassi, A., Melegaro, A., Gramegna, A., Ajelli, M., Merler, S., & ATS Lombardy COVID‐19 Task Force. (2021). Association of age with likelihood of developing symptoms and critical disease among close contacts exposed to patients with confirmed SARS‐CoV‐2 infection in Italy. *JAMA Network Open*, *4*, e211085.

13 Oberman, op. cit. note 5, p. 823.

14 Oberman, op. cit. note 5, p. 822; Carter, I. (1999). *A measure of freedom* (pp. 16–17). Oxford University Press; Kramer, M. H. (2003)*. The quality of freedom* (pp. 169–184). Oxford University Press.

15 I say ‘virus‐carriers’ because I accept Oberman's point that viruses are constraints imposed by other people acting as vectors (except in the case of patient‐zero, who has not been infected by another human agent).

16 Kramer, op. cit. note 14, pp. 196–197.

17 Carter, op. cit. note 14.

18 Ibid: 19.

19 Oberman, op. cit. note 5, p. 822.

20 Gao, Y., Bagheri, N., & Furuya‐Kanamori, L. (2022). Has the COVID‐19 pandemic lockdown worsened eating disorders symptoms among patients with eating disorders? A systematic review. *Zeitschrift fur Gesundheitswissenschaften*, *29*, 1–10; Hansen, S. J., & Menkes, D. B. (2021). What is driving the pandemic related surge in disordered eating? *British Medical Journal*, *374*, n2175.

21 Sova, C. (2021). *After a year of Covid, what lessons have we learned?* UN World Food Program USA. https://www.wfpusa.org/articles/after-one-year-of-covid-19-what-lessons-have-we-learned-about-hunger/

22 Booth, K. (2007). *Theory of world security*. Cambridge University Press.

23 Fischer, R. J., & Green, G. (2004). *Introduction to security* (p. 21). Butterworth‐Heinemann.

24 Berki, R. N. (1986). *Security and society: Reflections on law, order, and politics*. Palgrave Macmillan; Herington, J. (2012). The concept of security. In M. Selgelid & M. Enemark (Eds.), *Ethics and security aspects of infectious disease* (pp. 7–25). Ashgate.

25 Hunt, I. (1991). Freedom and its conditions. *Australasian Journal of Philosophy*, *69*(3), 288–230; Taylor, C. (1985). What's wrong with negative liberty. In C. Taylor (Ed.), *Philosophy and the human sciences: Philosophical papers* (Vol. 2, pp. 211–229). Cambridge University Press; Sen, A. (1988). Freedom of choice: Concept and content. *European Economic Review*, *32*(2–3), 269–294.

26 Berlin, op. cit. note 9, pp. 169, 178.

27 For instance, Gerald MacCallum argued that a meaningful notion of freedom must encompass both dimensions, so that freedom is really a triadic notion involving (1) an agent, who is (2) free from something (3) to do something. See MacCullum, G. (1967). Negative and positive freedom. *The Philosophical Review*, *76*(3), 312–334.

28 Isaiah Berlin, for instance, thought that the only true freedom is the negative one, because the positive ‘freedom to’ do things is inevitably defined by projections about what someone's ‘true self’ would want if that person were free from those factors (such as fears or other feelings) that compromise their rational wishes. This also risks providing support for illiberal and authoritarian policies. See Berlin, op. cit. note 9.

29 For instance, some scholars have defended, in response to Berlin, a minimalist notion of positive freedom compatible with liberalism and not necessarily leading to state prevarication. This is positive freedom defined in terms of ‘procedural conditions of autonomous decision‐making’. See, for example, Christman, J. (1991). Liberalism and individual positive freedom. *Ethics*, *101*, 343–359.

30 Taylor, op. cit. note 25, pp. 215–216.

31 Sen, op. cit. note 25.

32 Nozick, R. (1974). *Anarchy, state, and Utopia*. Basic Books.

33 Compare the case of a virus outbreak with the following case, suggested by an anonymous reviewer. Some terrorists have announced that they have placed traps in some buildings in London, but they have not said which buildings. If the traps are activated, buildings will be sealed and people trapped into the buildings. Intuitively, one would say that Londoners’ negative freedom has been limited by the traps, even if Londoners do not know which buildings have the traps and therefore entering a building is just a risk. The fact that I do not know that one of these traps is in the British Museum does not mean that I am free to go into the British Museum *and* leave. My ignorance does not make me free from the trap. Why would my ignorance of whether a virus will kill me ‐ that is, the fact that viruses are simply risks to me—mean that I am free from its consequences?

34 Kramer, op. cit. note 14, p. 194.

35 Steiner, H. (1994). *An essay on rights*. Blackwell.

36 Ferretti, M. P. (2016). Risk imposition and freedom. *Politics, Philosophy and Economics*, *15*(3), 261–279.

37 WHO. (2017). *Up to 650 000 people die of respiratory diseases linked to seasonal flu each year*. https://www.who.int/news/item/13-12-2017-up-to-650-000-people-die-of-respiratory-diseases-linked-to-seasonal-flu-each-year

38 Sometimes, the two terms are used figuratively in ways that do not match this distinction, for example, when we say that a flood is threatening people living near a river. However, it seems to me that we can assume that these are just anthropomorphizations of natural phenomena.

39 Bennett, J. (1998). *The act itself*. Oxford University Press.

40 Woollard, F. (2015). *Doing and allowing harm*. Oxford University Press.

41 Many languages do not have two different words for ‘freedom’ and ‘liberty’ in the way English language does, and even in English, the two terms are commonly used as synonymous, including in the philosophical and political literature. However, sometimes, a distinction is drawn along the lines that I have suggested here. See, for example, Pitkin, H. F. (1988). Are freedom and liberty twins? *Political Theory*, *16*(4), 523–552.

